# OMA orthology in 2021: website overhaul, conserved isoforms, ancestral gene order and more

**DOI:** 10.1093/nar/gkaa1007

**Published:** 2020-11-11

**Authors:** Adrian M Altenhoff, Clément-Marie Train, Kimberly J Gilbert, Ishita Mediratta, Tarcisio Mendes de Farias, David Moi, Yannis Nevers, Hale-Seda Radoykova, Victor Rossier, Alex Warwick Vesztrocy, Natasha M Glover, Christophe Dessimoz

**Affiliations:** SIB Swiss Institute of Bioinformatics, 1015 Lausanne, Switzerland; ETH Zurich, Computer Science, Universitätstr. 6, 8092 Zurich, Switzerland; Department of Computational Biology, University of Lausanne, 1015 Lausanne, Switzerland; SIB Swiss Institute of Bioinformatics, 1015 Lausanne, Switzerland; Department of Computational Biology, University of Lausanne, 1015 Lausanne, Switzerland; Center for Integrative Genomics, University of Lausanne, 1015 Lausanne, Switzerland; Department of Computational Biology, University of Lausanne, 1015 Lausanne, Switzerland; Department of Computer Science and Information Systems, BITS Pilani K.K. Birla Goa Campus, India; SIB Swiss Institute of Bioinformatics, 1015 Lausanne, Switzerland; SIB Swiss Institute of Bioinformatics, 1015 Lausanne, Switzerland; Department of Computational Biology, University of Lausanne, 1015 Lausanne, Switzerland; Center for Integrative Genomics, University of Lausanne, 1015 Lausanne, Switzerland; SIB Swiss Institute of Bioinformatics, 1015 Lausanne, Switzerland; Department of Computational Biology, University of Lausanne, 1015 Lausanne, Switzerland; Center for Integrative Genomics, University of Lausanne, 1015 Lausanne, Switzerland; Centre for Life's Origins and Evolution, Department of Genetics, Evolution and Environment, University College London, Gower St, London WC1E 6BT, United Kingdom; Department of Computer Science, University College London, Gower St, London WC1E 6BT, United Kingdom; SIB Swiss Institute of Bioinformatics, 1015 Lausanne, Switzerland; Department of Computational Biology, University of Lausanne, 1015 Lausanne, Switzerland; Center for Integrative Genomics, University of Lausanne, 1015 Lausanne, Switzerland; SIB Swiss Institute of Bioinformatics, 1015 Lausanne, Switzerland; Department of Computational Biology, University of Lausanne, 1015 Lausanne, Switzerland; Center for Integrative Genomics, University of Lausanne, 1015 Lausanne, Switzerland; SIB Swiss Institute of Bioinformatics, 1015 Lausanne, Switzerland; Department of Computational Biology, University of Lausanne, 1015 Lausanne, Switzerland; Center for Integrative Genomics, University of Lausanne, 1015 Lausanne, Switzerland; SIB Swiss Institute of Bioinformatics, 1015 Lausanne, Switzerland; Department of Computational Biology, University of Lausanne, 1015 Lausanne, Switzerland; Center for Integrative Genomics, University of Lausanne, 1015 Lausanne, Switzerland; Centre for Life's Origins and Evolution, Department of Genetics, Evolution and Environment, University College London, Gower St, London WC1E 6BT, United Kingdom; Department of Computer Science, University College London, Gower St, London WC1E 6BT, United Kingdom

## Abstract

OMA is an established resource to elucidate evolutionary relationships among genes from currently 2326 genomes covering all domains of life. OMA provides pairwise and groupwise orthologs, functional annotations, local and global gene order conservation (synteny) information, among many other functions. This update paper describes the reorganisation of the database into gene-, group- and genome-centric pages. Other new and improved features are detailed, such as reporting of the evolutionarily best conserved isoforms of alternatively spliced genes, the inferred local order of ancestral genes, phylogenetic profiling, better cross-references, fast genome mapping, semantic data sharing via RDF, as well as a special coronavirus OMA with 119 viruses from the Nidovirales order, including SARS-CoV-2, the agent of the COVID-19 pandemic. We conclude with improvements to the documentation of the resource through primers, tutorials and short videos. OMA is accessible at https://omabrowser.org.

## INTRODUCTION

Genes which are related through speciation are called orthologs, as opposed to paralogs, which are related through duplication (1). This distinction is useful in a wide range of contexts, including phylogenetic tree inference, protein function prediction, or whole genome alignment (reviewed in 2).

For over 15 years, the OMA (‘Orthologous Matrix’) database has elucidated orthologs among complete genomes across the entire tree of life (3–6). In this update paper, we report on the most recent developments, including new and updated species, website overhaul, improved isoforms handling, improved crosslinks, improved gene ontology function predictions, phylogenetic profiling, fast genome mapping, improved documentation, as well as a coronavirus OMA database.

## NEW AND UPDATED SPECIES

The number of species in OMA has steadily grown and now stands at 2326. In particular, in the past three years, we added 23 protists, 11 plants, 45 fungi, 14 fishes, 4 birds and 25 mammals. We have added one allopolyploid species (*Xenopus lavis)* to the existing ones (*Triticum aestivum*, *Gossypium hirsutum*, *Brassica napus*), for which we also compute homoeologs, which are the related genes resulting from allopolyploidisation (7). In addition to new genomes, we update the genomes of model species at each release.

The prioritisation of new and updated genomes is mainly driven by our users, so we invite researchers to provide specific suggestions by filling in the following form: https://omabrowser.org/suggest.

## NEW ORGANISATION AROUND GENES, GROUPS AND GENOMES

**Figure 1.**
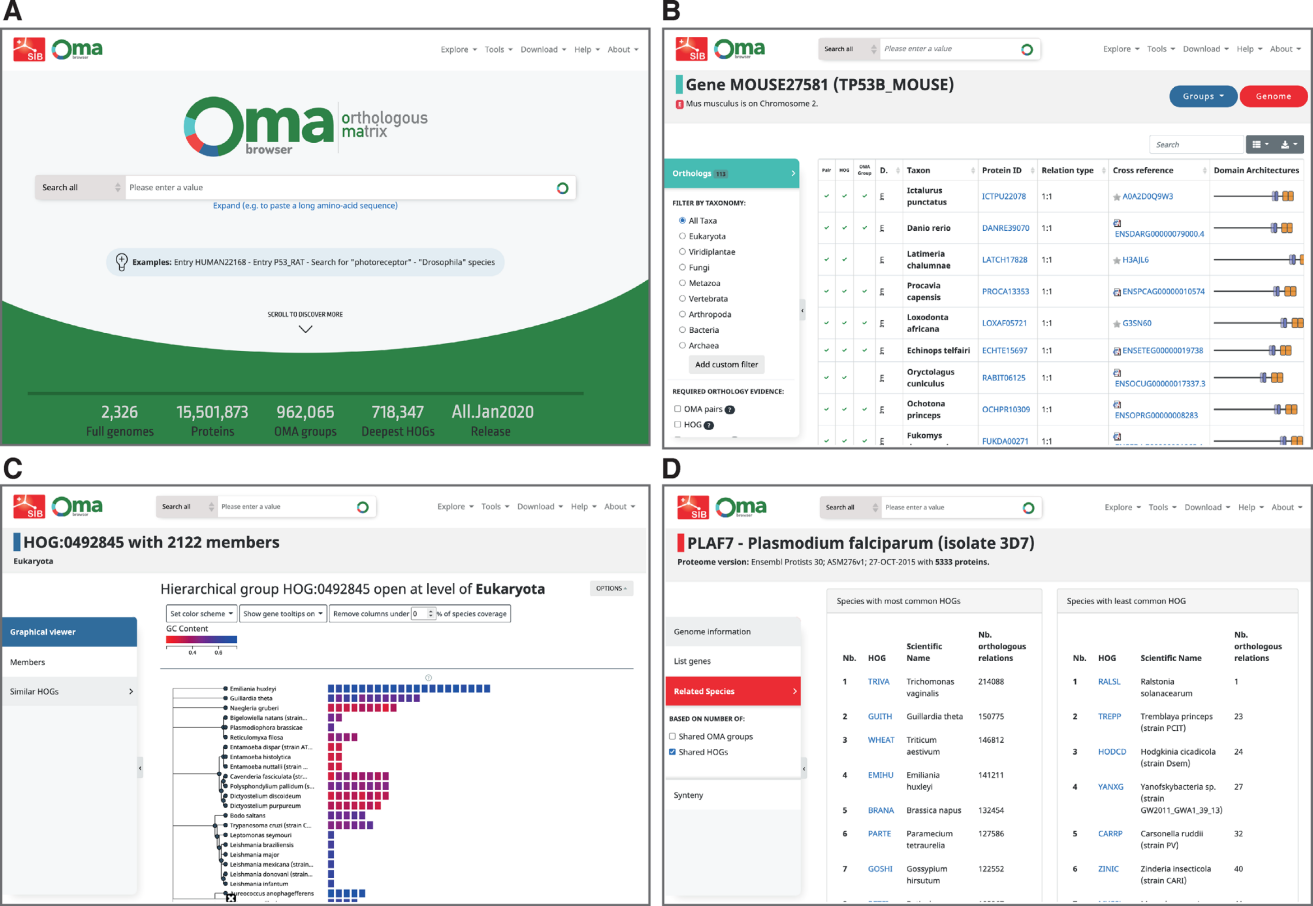
New OMA Browser website, with a new landing page (**A**), and the database part organised in genes (**B**), groups (**C**) and genomes (**D**).

The OMA browser design and architecture have been overhauled. The database part of the browser is now articulated around the three major data types: genes, groups and genomes (Figure 1).

Gene-centric pages, recognisable by their light blue colour theme, provide information with respect to a particular gene, including sequence, cross references, functional annotations, as well as evolutionary information. The table of orthologs has been improved to include the evidence supporting each prediction: the browser reports whether a particular pair is predicted to be orthologous based on pairwise analyses, by virtue of being in the same OMA group, and/or by being in the same Hierarchical Orthologous Group (HOGs—nested groups of genes which have descended from a common ancestral gene in a given clade of species). More details on the various types of pairwise orthologs are provided in a recent primer (8). Using the side menu, users can filter the list to particular lines of evidence, or particular taxonomic clades. Another novelty is the table of pairwise paralogs, which are derived from the HOGs only (since neither pairwise comparison nor OMA groups reliably induce paralogy).

Group-centric pages, recognisable by their dark blue theme, are of two subtypes: OMA groups, which are groups in which every gene is orthologous to every other one, and HOGs, which provide a formal way of defining families and subfamilies, and provide a model of the proteomes of ancestral genomes. More details on the differences between OMA Groups and HOGs and their uses are provided in the primer (8). Either group type provides a list of members and the ability to look for closely related groups.

Finally, genome-centric pages, recognisable by their red theme, provide information on the underlying species, a list of all genes associated with them, a list of closely related genomes in OMA, and access to the pairwise global synteny viewer introduced earlier (6).

## ISOFORMS HANDLING

Eukaryotic species, especially vertebrates, use alternative splicing, by which a diversity of protein sequences can originate from a single gene by varying combinations of its exons (9). Most orthology resources select *a priori* one reference (‘canonical’) isoform to be used for orthology inference, usually the longest one. By contrast, OMA keeps multiple candidate isoforms for the all-against-all alignment phase, and selects as reference the isoform which has the best matches across all species—which can be thought of as the most evolutionarily conserved isoform. Interestingly, in the current release, the reference isoform selected by OMA is *not* the longest one for 48.6% of all genes with more than one isoform.

While this reference isoform procedure has been part of the OMA algorithm since its inception, we have improved our reporting of isoforms in the Browser. First, we list all isoforms in a table accessible from the gene-centric view, with lengths, exon structure and indication of the reference isoform selected by OMA. Second, starting from now, we will include in the Browser all isoforms annotated in the input genomes, even those which we do not consider as candidate isoforms in the all-against-all computations (to save computations, we disregard isoforms which are covered by candidate isoforms in at least 90% of their length). This change will roll out as we update and add new genomes to OMA.

Additionally, isoform data is now available through the programmatic access (REST API), under the protein section. The query takes as an input the identifier of the gene, and outputs all isoforms, including their identifiers and genomic coordinates, and specifies which isoform was selected as reference.

## CROSS-REFERENCES AND SEARCH ENGINE

The OMA Browser is part of a rich ecosystem of bioinformatic resources. We import genomic and functional data from various different sources, notably Ensembl (10), UniProt (11), RefSeq (12), Gene3D (13) and HGNC/VGNC (14). Conversely, OMA orthologs are integrated by various resources, including UniProt, HGNC/VGNC, GenBank (15), the Alliance of Genome Resources (16), and Bgee (17). Furthermore, many OMA users also combine OMA orthologs with other resources. Cross-references to other resources are thus critical.

We have extended our cross-references in two ways. First, we now provide cross-references to additional resources, including STRING (18), Bgee (17) and Swiss-Model (19). The second way pertains to the mapping criteria. Until now, we have adopted stringent requirements to establish cross-references. For instance, to introduce a cross-reference to UniProt or RefSeq, we have required both exact species and exact sequence matching. However, this has occasionally caused confusion to some of our users, who can fail to find a gene or protein of interest in OMA due to minute differences in the sequence (a problem which particularly affects well-curated Swiss-Prot entries, which often include sequence corrections). To address this problem, we have introduced an additional mapping procedure which relaxes the exact match requirement. When a cross-reference is inexact, we still provide it but warn users of this fact on the browser. The mapping mode (strict or tolerant) is also exposed in the relevant REST APIs. For UniProtKB, RefSeq, and Entrez Gene alone, also accepting inexact mapping resulted in a 39% increase in cross-references—to a total of 31.9 million.

In addition, we have also improved the search engine in various ways. Auto-completion has become case insensitive, and indexes more information (e.g. gene description). The search result page presents results according to the new organisation in three kinds of data (gene, groups, genomes). Search speed has also improved thanks to better indexing on the server side.

## TOWARD RECONSTRUCTING THE SYNTENY OF ANCESTRAL GENOMES

Reconstructing the ancestral genome order is a powerful approach for better understanding the relationship between extant genes across species as well as the processes that generated these patterns (20–24). Since each HOG corresponds to a particular ancestral gene, reconstructing the ancestral gene order amounts to inferring the order of HOGs defined for a particular node of the species tree. To this end, we have developed a procedure which propagates gene adjacencies across the species tree, bottom up. In essence, at each ancestral node, we infer HOG adjacencies by merging information from the immediate descendants, using the pyHam library (25) to map genes between each parent and child species. Inconsistencies, which can be the result of genomic rearrangements or assembly errors, can be resolved using the majority rule. Note that at this stage, we do not attempt to reconstruct a global order of HOGs, and merely report the propagated adjacencies as likely neighbours in the HOG pages and the REST API.

**Figure 2.**
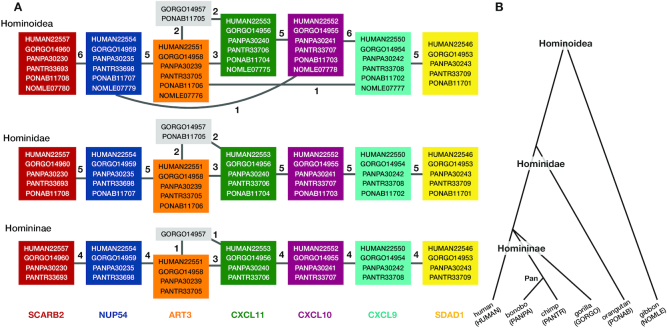
Example ancestral gene synteny reconstruction. (**A**) We reconstruct the adjacencies (edges) among HOGs (coloured boxes) defined for each of the three key ancestors of the human, chimpanzee, bonobo, gorilla, orangutan, and gibbon (**B**). At all levels, some HOGs have more than two adjacencies. However, accounting for the weights (majority rule), we can infer the best path through the graph which would omit the unnamed HOG present in gorilla and orangutan.

Consider for example, the neighborhood of the cytokine *CXCL11* gene within the Hominoidea clade, which encompasses humans, chimpanzees, bonobos, gorillas, orangutans, and gibbons (Figure 2). Gene adjacencies are depicted as edges, with weight according to the amount of support given by each species lower in the tree. Five species support the adjacency of *CXCL11* to *CXCL10* in the last hominoidea ancestor, giving it a weight of 5. The other *CXCL11* neighbour is likely *ART3*, but we can also see that gorilla and orangutan support an adjacency to an unnamed HOG (depicted in gray in Figure 2), yet this is less strongly supported than the connection to *ART3* shown by chimp, bonobo and human. Nevertheless, the remaining adjacencies are highly consistent within the Hominoidea, making it possible to infer the order of most ancestral genes found in this neighbourhood.

## IMPROVEMENTS TO THE GRAPHICAL HOG VIEWER iHam

To explore the relationships among related HOGs defined at each taxonomic level, we previously introduced the graphical viewer iHam (25). To visualise the evolutionary dynamics of a gene family, one useful feature is to colour genes, represented as squares, according to gene length or average GC content. We have added a colouring option to visualise the number of exons, as well as Gene Ontology (GO) function similarity. Furthermore, GO annotations associated with each gene are provided in the tooltips. Since HOGs can have many hundreds or even many thousands of members, we devised an efficient way of computing similarities, by using the MinHash approach (26) for estimating the Jaccard distances (27), which thus avoids costly all-against-all comparisons.

Consider for instance the exon number view for the HOG of the gene encoding human RNA polymerase II subunit G (*POLR2G*) at the mammalian level. Most mammals have a single gene with 4–10 exons, but the little brown bat *(Myotis lucifugus)* also contains an extra copy with a single exon, suggesting that the gene encoding G1P1C7 (UniProt ID) is a retrogene (resulting from the reverse transcription of a spliced mRNA). The local synteny view of this gene provides corroborating evidence that the additional, single-exon gene does not have any conserved neighborhood in other species, which is typical for a lineage-specific gene retrocopy.

## PHYLOGENETIC PROFILING

Genes that are involved in the same biological processes tend to be jointly retained or lost across evolution. Thus, such co-evolution patterns can be used to infer functionally related genes—a technique known as ‘phylogenetic profiling’ (28). Recently, we introduced HogProf, an algorithm to efficiently identify similar HOGs in terms of their presence or absence at each extant and ancestral node in the genome taxonomy, as well as the duplication or loss events on the branch leading to that node (29). This functionality has been added to the OMA browser, making it possible, starting from any HOG, to identify similar HOGs using similar phylogenetic patterns.

The functionality is available from HOG pages, under the ‘similar HOGs’ tab, and complements other ways of identifying similar HOGs based on domain architecture or on sequence similarity. Of note, the visual representation of the profile available on the web interface only shows the extant species covered by the query and returned HOGs. The actual profile similarities are calculated between the set of taxonomic nodes where ancestral presence was inferred along with extant species, as well as the set of ancestral duplications and losses shared between HOGs (29). Finally, phylogenetic profiles are also retrievable via the REST programmatic interface (under HOGs methods).

Genes involved in the eukaryotic cilium have been the subject of several phylogenetic profiling studies, in part due to their particular evolutionary histories (30). The phylogenetic profiling tool integrated in the OMA Browser allows easy finding of genes bearing this signature. Starting from the HOG encompassing human gene *IFT52*, one of the components of the intraflagellar transport machinery essential for ciliogenesis, we inspected the ten closest HOGs returned by OMA. All of them had known association with the cilium: seven are other components of the intraflagellar transport machinery (*IFT56*, *IFT80*, *IFT122*, *IFT140*, *IFT157*, *IFT172*, *WDR19*), two are part of the dynein complex required for correct intraflagellar transport (*DYNC2LI1*, *DNALI1*), and one (*BBS5*) is part of the BBSome complex, required for ciliogenesis.

## FAST GENOME MAPPING

The ever accelerating pace of genomic sequencing is such that OMA can only focus on a subset of all public genomes available. Many will remain absent from orthology databases such as OMA. Thus, there is an interest in efficiently transferring knowledge from orthology databases to genomes provided by the user. One solution is given by OMA standalone (31), which can efficiently combine OMA and custom data. In the last update paper, we also introduced GO function prediction tool based on fast mapping to the closest sequence (6). Since then, we have improved the performance of the fast sequence mapper. This method relies on a *k*-mer index, which is built from a suffix array—a sorted array of all suffixes of a particular string (32). This index is then used to perform an initial *k*-mer mapping, before refining the order of matches with a small number of Smith-Waterman alignments. The index enables constant-time lookup for each *k*-mer, meaning the time-complexity of the initial filtering is relative to the length of the query sequence. Users can retrieve the closest match for all input sequences—either across all of OMA, or in a target genome. Alternatively, it remains possible to infer GO annotations based on the closest sequence, as introduced in the previous update paper (6).

## SEMANTIC INTEROPERABILITY AND FEDERATED QUERIES BASED ON RDF

Recent years have witnessed an increasing adoption of Resource Description Framework (RDF) among bioinformatics databases to model their data, as evidenced by the YummyData monitor (33), which currently highlights more than 65 biological and biomedical resources with SPARQL endpoints for querying their data (SPARQL is the main query language for RDF). The attractiveness of using RDF and SPARQL for bioinformatics databases can be explained by three main factors: (a) the virtuous cycle of adopting a common data syntax and model that leverages data interchange on the Web; (b) the SPARQL 1.1 specification makes it possible to run federated queries, which allow bioinformaticians to jointly retrieve data from multiple resources in one single query and (c) the existing growing number of RDF-based ontologies, controlled vocabularies, and taxonomies in life sciences. For example, the Bioportal, a repository of biomedical ontologies, contains >850 ontologies (34).

OMA has been available in RDF for several years, in addition to other interfaces, namely the web browser, bulk file downloads and the REST API (and associated R and Python omadb libraries) (35). We have made several improvements to the RDF representation of OMA data. In the context of the *Quest for Orthologs* consortium, we co-authored the improvement and release of the ORTH ontology version 2 (36). ORTH is an ontology to describe and structure orthology data in RDF and related information such as paralogy. This ontology was designed to semantically harmonize the data from different QfO consortium databases. Semantic harmonization is the process of consolidating different data sources and representations into a form where portions of data share meaning (37). The OMA SPARQL endpoint is now fully compatible with the newest ORTH version. The endpoint is also part of a federated architecture for biological data integration. Several question templates addressed over multiple databases are available (38).

As mentioned above, SPARQL is of particular interest to retrieve RDF data across multiple resources. In the SwissOrthology portal (https://swissorthology.ch), we provide consensus orthology calls from OMA and OrthoDB (39) using federated SPARQL queries in the backend. More technical details on how to retrieve orthologs across multiple orthology resources using SPARQL are available in a recent tutorial (40).

## CORONA OMA BROWSER

The immense speed of spread and cost, in both life and economy, of the novel SARS-CoV-2 virus has made it an intense focus of scientific research. To accommodate this vast interest in the SARS-CoV-2 strain and its relation to other single-stranded RNA viruses, we created the Corona OMA Browser (https://corona.omabrowser.org). This website provides all the same functionalities as the non-viral genome OMA Browser, including access via a REST API. The aim of the Corona OMA Browser is to be a resource that assists researchers in gaining insight into the functional and evolutionary aspects of coronaviruses. It helps unravel the phylogenetic relationships among 119 species from the *Nidovirales* order, including 82 *Coronaviridae* affecting numerous mammals and birds. The viral proteomes were obtained from UniProtKB reference proteomes, and the corresponding DNA sequences were obtained from the European Nucleotide Archive (41).

Using the interactive Browser, it is straightforward to identify the species included in the database and to visualize the domain architecture and functional annotations of viral proteins. The Browser can be used to obtain and compare viral gene families with Hierarchical Orthologous Groups (HOGs). Further, one can obtain orthology-informed functional annotations on the coronavirus genes. New gene sequences can be mapped to existing gene families in the browser to infer the function of proteins. Using the ‘Export Marker Genes’ option, genome-wide phylogenomic inference can be performed (42). In addition, the Corona OMA Browser facilitates the exploratory analysis of patterns of gene duplication/loss using HOGs and iHam visualisation. The Browser also aids the exploration of the conserved local genomic neighbourhood (synteny) across the available viral species. Ultimately, all these functionalities make the Corona OMA Browser a powerful, user-friendly tool which could complement and further the research of coronaviruses.

## IMPROVED DOCUMENTATION

The OMA resource provides not only a wealth of orthology information, but also many other tools to help facilitate user-side analyses of the OMA data. We have steadily improved the documentation of OMA to explain confusing concepts, show how to extract orthology data in a number of ways, or to use the extracted OMA data as a launching point for downstream analyses. A few months ago, we launched a collection of tutorials entitled the ‘OMA Collection’, with the aim to introduce concepts and showcase analyses and applications enabled by OMA in an hands-on manner (43).

The OMA primer (8) serves as a starting point for new OMA users, and goes into detail about the gene- and group-centric information we provide in OMA. We focus on the different types of orthologs, how the OMA algorithm infers them, and conceptual and practical differences between them. Complementary to that, we also produced short introductory videos introducing the concept of HOGs (https://youtu.be/5p5x5gxzhZA) and how these can be visualised and interactively explored in OMA (https://youtu.be/6eAoamP7NLo).

For more advanced users, we wrote a tutorial on the R and Python OMA libraries (35), as well as the aforementioned SPARQL orthology tutorial (40). We also produced a video on how to use a SLURM scheduler to run OMA Standalone (31) in parallel on custom data (https://youtu.be/a1FqwGZ0WV4).

As for applications, we wrote a tutorial on how to build phylogenetic species trees with OMA (42). In particular, we show how to select Orthologous Groups as phylogenetic marker genes, build a concatenated supermatrix, and run external software to make an alignment and tree.

In all the above articles, videos, tutorials, and protocols, we use publicly available software and provide scripts, code, practical examples, and plenty of explanations in order to facilitate the use of OMA in user analyses.

## DATA AVAILABILITY

OMA data are available in various formats, including the interactive website, flat files, RDF, REST API, R and Python libraries. OMA is licensed under a Creative Commons Attribution-Share Alike 2.5 License. The underlying sequences and annotations may be subject to third-party constraints. Users of the data are solely responsible for establishing the nature of and complying with any such intellectual property restrictions.
